# Reproductive health services utilization and its associated factors among secondary school youths in Woreta town, South Gondar, North West Ethiopia: a cross sectional study

**DOI:** 10.1186/s13104-019-4129-z

**Published:** 2019-02-15

**Authors:** Addisu Taye Abate, Aynalem Adu Ayisa, Tesfamichael G/Mariam W/Mariam

**Affiliations:** 1 Department of Medical Nursing, School of Nursing, College of Medicine and Health Sciences, University of Gondar, Gondar, Ethiopia; 2 Department of Surgical Nursing, School of Nursing, College of Medicine and Health Sciences, University of Gondar, Gondar, Ethiopia

**Keywords:** Reproductive health service, Students, Utilization, Youth, Reproductive health, Ethiopia

## Abstract

**Objective:**

The aim of this study was to assess reproductive health service utilization and its associated factors among secondary school students in Woreta town, South Gondar, North east Ethiopia 2018.

**Result:**

Out of the total 376 selected students, 345 were participated in the study with a response rate of 94%. Of these respondents, 85 (24.6%) of them utilized at least one reproductive health services in the past 1 year. Voluntary testing and counseling for HIV/AIDS and Family planning services were utilized by 47 (55.3%) and 43 (50.6%) of youths respectively. Being grade 11–12 (AOR = 5.299, 95% CI 2.019, 13.912, P = 0.001) and closeness of the service facility to their home (AOR = 2.76, 95% CI 1.168, 6.525, P = 0.021) were significantly associated with students’ reproductive health service utilization. This low service utilization might make students prone for different reproductive health risks; which in turn can increase school dropout rate, and has impact on individual’s future life as well as the country’s youth health policy from meeting its goal. Therefore, it needs a great effort and attention of all the concerned bodies including parents, school staffs, and health professionals to improve the service utilization in schools.

## Introduction

Reproductive health (RH) is a state of complete physical, mental and social well-being relating to the reproductive system; and not merely the absence of disease or infirmity [1]. According to World Health Organization (WHO), youths are described as a young person between the age group of 15 and 24 years [2]; those make up about 20% of the world’s population [3]; of whom, 85% lives in developing countries [4]. Youths considered being the hope for future of one country and health condition of each individual as well as behavioral formation made during this period has also impact on development of the country [1, 5]. Despite this, they have unique reproductive health risks of unplanned pregnancies and childbearing; sexually transmitted infections (STIs) including Human Immuno Deficiency Virus (HIV) and unsafe abortion [6–9]. However, the access to and utilization of Reproductive Health services (RHS) is a primary concern for Youths surrounding the promotion of reproduction health and rights [10].

In Ethiopia, more than 1/3 of the population is found between the age of 10-24, in which they are the most vulnerable to a range of reproductive health problems as a result of premarital sexual activities [11]. For Youths to effectively transit to adulthood, they need to be provided with factual, accessible and affordable reproductive health information and services [3, 12]. As a response, the Ethiopian government, along with a number of international Non-Governmental Organizations (NGOs), has been supporting activities including the scale-up and institutionalization of Youth Friendly Services through intensive capacity building at all levels of the health system [13, 14]. However, the effects of all the efforts have not been felt across the Ethiopian learning institutions, as it is evidenced by persistent reproductive health problems and challenges of the youths such as unwanted pregnancy and its consequences: the rate of abortion among students was found to be 65 per 1000 women, making it threefold of the national rate of abortion for Ethiopia (23/1000 women aged 15–44); increasing prevalence of STIs including HIV/AIDS (19.5%) [4, 15, 16]; unmet need for family planning and over a quarter of all Ethiopian pregnant youths and adolescents feel that their pregnancies are mistimed; high rates of delivery-related complications as well as a subsequent drop-out of school for many young girls [17, 18].

Secondary school students are the most vulnerable group for RH problems due to their inclination to be engaged in risky sexual behavior [16]. Additionally, the environment itself expose students to greater opportunities and circumstances for engaging in risky behaviors related to RH [19]. Furthermore, attracting youths to the clinical services has remained a challenge; however, there was a need to improve the health seeking behavior of these age groups [20]. Despite these, the response to deliver RHS for students has been fragmented and Ethiopian schools are limited in delivering health related services for their students. As a result significant proportion of their students are developing RH related problems [21]. Although such studies are important in resource-limited areas, to give the present image of reproductive health service related issue of the students, there is no study conducted in Woreta town secondary school students regarding utilization of RHS; and the available literatures in Ethiopia were limited in addressing factors that influence utilization of RHS among secondary school students. Therefore, this study aimed to assess Utilization and factors affecting RHS among secondary School Students in Woreta town, North West Ethiopia.

## Main text

### Study design, area and period

Institutional based cross-sectional study was conducted from April 20 to May 15/2018 among secondary school students at Woreta town, North West Ethiopia.

### Sampling and participation

The source population were all youths attending secondary school at Woreta town during 2018 academic year. All students who are 15–24 years old attending their secondary school within the town during the study period were included; whereas, those students who are seriously ill at time of data collection were excluded. The sample size was determined by using single population proportion formula by considering the following assumption: 32% proportion of RHS utilization [22], 95% confidence interval, 5% margin of error and 5% for possible non-response. Based on these, a total of 376 students was taken as a final sample size. In Woreta town, there was only one secondary school having a total of 3213 students attending grade 9–12 in 2018 and these are taken as a study population. The sample size was proportionally allocated to each grade and computer generated simple random sampling technique was used to select the study participants from each grade by using the list of all students in each grade as a sampling frame.

### Data collection tool and procedure

Data collection was performed by Four B.Sc. nurses, who were properly trained for 2 days regarding the tool and the procedure (on the objective of the study, how to approach the students, how to administer and collect the questionnaires timely). The questionnaire was first prepared in English and then translated to local language, Amharic, and re-translated back to English to ensure consistency. The tool was tested on 18 secondary school students at Debretabore city and relevant modifications were done before the actual data collection period. Data on socio demographic characteristics, health care system factors/accessibility of RHS facility and reproductive health service utilization were collected by using structured self-administered questionnaire [23].

### Data processing and analysis

After the questionnaires checked visually for completeness, entered into Epi Info version 7 and exported to Statistical Package for Social Sciences (SPSS) version 23 for analysis. Descriptive statistics including frequencies, percentages, summary statistics like mean and standard deviation were computed to describe the study participants. Binary logistic regression was employed to see the crude significant association of each variable to the outcome variable, RHS utilization. Then variables with *P* value < 0.2 in bi-variable logistic regression analysis were again entered into multivariable logistic regressions to see the independent effect of each explanatory variables with the outcome variable. Finally, variables having P-value < 0.05 in multivariate analysis were considered as statistically significant predictor of RHS utilization based on adjusted odd ratio (AOR) with 95% confidence level.

### Operational definition

#### Reproductive health service utilization

Utilization of at least one of reproductive health services (HIV testing and counseling, STI screening and treatment, family planning counseling and contraceptive use, abortion service, and perinatal services) within the last 1 year [23]. Accessibility of RHS facility (geographical accessibility): in this study accessibility was measured in terms of estimated distance of RHS facility from their home [24].

### Results

#### Socio-demographic characteristics of participants

A total of 345 students were participated in the study with a response rate of 94%; out of this 184 (53%) were females with a mean age of 17.8 (SD ± 1.76) and almost half of them 179 (51.8%) were grade 11 and 12. 277 (80.3%) of student stated as they have discussed sex related issues with their parents in the past 1 year and 236 (68.4%) of them get pocket money for daily expenses (Table 1).

**Table 1:** Socio-demographic characteristics of secondary school youths in Woreta town, South Gondar, Ethiopia 2018 (n = 345)

Variable	Category	Frequency	Percentage (%)
Sex	Male	161	46.7
	Female	184	53.3
Age	15–19	286	82.9
	20–24	59	17.1
Grade level	Grade 9–10	166	48.2
	Grade 11–12	179	51.8
Religion	Muslim	23	6.7
	Protestant	5	1.4
	Other	1	0.3
Ethnic group	Amhara	342	99.1
	Oromo	2	0.6
	Tigre	1	0.3
	Other	0	0
With whom do you usually live	With my father and mother	284	82.3
	With mother only	29	8.4
	With father only	7	2.0
	With relatives	6	1.7
	With friends	10	2.9
	Alones	9	2.6
How often did you discuss sex related issues with your parents	In the past 3 months	30	8.7
	In the past 6 months	38	11.0
	In the past 1 year and above	277	80.3
Parents education level	Don’t read and write	72	20.9
	Reade and write only	142	41.2
	Primary school	46	13.3
	Secondary school	30	8.7
	Diploma	47	13.6
Marital status of the mother and father	Currently not live with parents	8	2.3
	Together	284	82.3
	Separate	14	4.1
	Divorced	15	4.3
	Widows	32	9.3
Do you get pocket money for daily expenses	Yes	236	68.4
	No	109	31.6

##### Utilization of RH services by secondary school youths

**Fig. 1:**
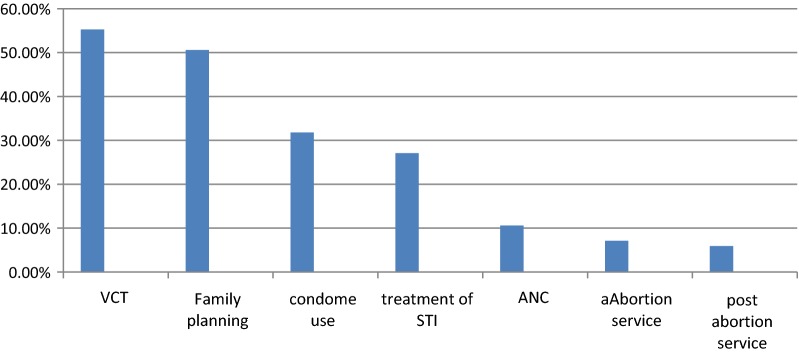
Utilization of reproductive health service among secondary school students in Woreta town, South Gondar, Ethiopia, 2018 (N = 345)

From the total of 345 school youths, 85 (24.6%) of them utilized reproductive health services in the past 1 year. Regarding the components of RHS voluntary testing and counseling of HIV, family planning and condom were utilized by 47 (55.3%), 43 (50.6%) and 27 (31.8%) youths respectively (Fig. 1).

##### Factors associated with utilization of RHS

On the bivariate analysis, sex, grade level of the youths, having pocket money for daily expense, parental educational status and nearness of RHS facility to the participants’ home were identified to be significantly associated with youths’ RHS utilization. During multivariable logistic analysis grade level of participants and closeness of RHS facility to their home were continued to be significant. The likely hood of RHS utilization was 5.299 times more likely in grade 11–12 (AOR = 5.299, 95% CI 2.019, 13.912, P = 0.001) when compared to grade 9–10. Youths with RHS facility near to their home were 2.76 times more likely to utilize the service than far to their home (AOR = 2.76, 95% CI 1.168, 6.525, P = 0.021) (Table 2).

**Table 2:** Factors associated with RH services utilization among secondary school students in Woreta town, South Gondar, Ethiopia 2018

Variables	RH service utilization (%)		COR (95% CI)	P-value	AOR (95% CI)	P value
	Yes	No				
Sex						
Female	51 (60%)	133 (51.2)	1.432 (0.871, 2.335)	0.157	0.435 (0.177, 1.09)	0.70
Male	34 (40%)	127 (48.8)	1.00		1.00	
Grade level						
11–12	*44 (51.8%)*	*41 (48.2%)*	*3.3.27 (1.913, 10.43)*	*0.001*	1.00	
9–10	41 (48.2%)	*125 (48.2%)*	*1.00*		*5.299 (2.019, 13.91)*	0.001*
Do you get pocket money for daily expense?						
Get money for daily expense	65 (76.5%)	171 (65.8%	0.591 (0.337, 1.038)	0.067	2.595 (0.153, 0.856)	0.50
Don’t get money for daily expense	20 (23.5%)	89 (34.2%)	1.00		1.00	
Is RH service near to your home?						
Near to home	*52 (61.2%)*	*130 (50%)*	*1.576 (0.956, 2.596)*	*0.074*	*2.76 (1.168, 6.525)*	0.021*
Far to home	33 (38.8%)	130 (50%)	1.00		1.00	
Educational status of parent						
Not read and write	21 (24.7%)	51 (19.6%)	1.00		1.00	
Read and write	39 (45.9%)	103 (39.6%)	1.087 (0.581, 2.037)	0.793	2.021 (0.656, 6.22)	0.22
Primary education	7 (8.2%)	39 (15.0%)	2.294 (0.886, 5.994)	*0.087*	2.58 (0.653, 10.47)	0.18
Secondary education	9 (10.6%)	21 (8.1%)	0.961 (0.378, 2.439)	0.933	1.434 (0.284, 8.41)	0.69
Diploma and above	8 (9.45)	39 (15.0%)	2.007 (0.804, 5.011)	*0.135*	4.033 (0.901, 18.052)	0.68
Convenient place for RH service						
Anywhere out of the resident area	51 (60.0%	140 (53.8%)	0.183 (0.024, 1.421)	0.104	0.409 (0.04, 4.165)	0.45
In the center of the town	22 (25.9%)	67 (25.8%)	0.203 (0.25, 1.625)	0.133	0.27 (0.027, 2.891)	0.285
At the end of the town	11 (12.9%)	38 (14.6%)	0.230 (0.07, 1.943)	0.177	0.147 (0.011, 1.91)	0.144
In the school	1 (1.2%)	15 (5.8%)	1.00		1.00	

NB: variables having a (P < 0.2) in bi variable (unadjusted) analysis included in the multivariable (adjusted) analysis

*COR* Crude odd ratio, *AOR* adjusted odd ratio

* Statistically significant at P-value < 0.05

### Discussion

The overall utilization of RHS among secondary school youths in Woreta was found to be 24.6%, (95% CI 19.8% - 29%); which is in line with a study conducted in Nekemet [25], Madawalabu university [26], Mekele [27] and Mechakel, East Gojjam [28]; in which (21.2%, 27.7%, 23%, and 21.5%) of participants utilized RHS respectively. However, this finding is lower than studies conducted in Harare, Bahir Dar, Awabel and Hadiya Zone, Ethiopia, which showed that (63.8%, 32%, 41.2% and 38.5%) of participants utilized at least one of the reproductive health service in the past 1 year [22, 29–31] respectively. The possible reason for this difference might be due to the participants’ socio-demographic characteristics, time reference used in the definition of RHS utilization and socio economic variation. Furthermore, this discrepancy might be also due to differences in the availability and accessibility of reproductive health facilities and youth centers within the school.

In the current study, the commonly utilized RHS component was voluntary testing and counseling service (55.3%) which is consistent with the findings of studies done in Harrar [29] and Mecha District, North West, Ethiopia [32]; in which 52.8% and 60.2%  % of participants utilized voluntary testing and counseling service respectively. But, differ from studies conducted in Hawassa, Madawalabu, Kenya and Nigeria, in which family planning was the commonly utilized RHS [26, 33–35] respectively. This discrepancy might be due to socio demographic variation like difference in age and maturation. For example, in Hawassa [33], and Madawalbu [26], the study was conducted among University students whose age and maturation are expected to be relatively higher than secondary school students. In addition, family and community influences are relatively low; but high peer influence and risk perception in university students than secondary school students. So, all these things might made difference in RHS utilization.

In this study, grade eleven and closeness of RHS facility to their home were found to be significantly associated with RHS utilization among school youths. In this regard, grade eleven and twelve students were Five times more likely to utilize RHS (AOR = 5.299, 95% CI 2.019, 13.912, P = 0.001) compared to grade nine and ten students. This finding is supported by studies conducted in Addis Ababa, Ethiopia and Kenya where difference in educational level is significantly associated with RHS utilization [22, 34]. This might be due to the fact that becoming more disclosure for RHS information and secondary behavioral change as the grade level increases Similarly, Youths with RHS near to their home were 2.76 times more likely to utilize the service than Youths with RHS facility is far to their home (AOR = 2.76, 95% CI 1.168, 6.525, P = 0.021).

### Conclusion

Reproductive health service utilization among secondary school students in Woreta town found to be low. This might make students prone for different problems related to reproductive health; which in turn can increase school dropout rate. The most frequently utilized RHS component was voluntary testing and counseling followed by family planning service. In this study grade 11–12 and closeness of facility near to their home were significantly associated with the service utilization. Overall, the finding requests the attention and effort of all concerned bodies including parents, school staffs, and health professionals to improve the service utilization in schools. Furthermore, a study supported by qualitative techniques should be done including youths’ parents and service providers’ opinion.

## Limitation

Since the study was institutional based and which was also confined to the public school, generalization of the findings to the general youth population is limited. In addition, the study examines personal and sensitive issues through a quantitative study design with self-administered questionnaire, which may have subjectivity.

## Abbreviations

AIDS: acquired immuno-deficiency syndrome
ANC: anti natal care
AOR: adjusted odd ratio
CL: confidence level
COR: Crude odds ratio
Epi info: statistical package for epidemiological information analysis
FP: family planning
HIV: human immuno-deficiency virus
OR: odds ratio
PNC: post natal care
RH: reproductive health
STI: sexual transmitted infection
STD: sexual transmitted disease
SPSS: Statistical Package for Social Science
University of Gondar: University of Gondar
VCT: Voluntary Counseling and Test
WHO: World Health Organization
